# Intra-Uterine Medication

**Published:** 1871-02

**Authors:** 


### INTRA-UTERINE MEDICATION.

Prof. E. R. Peaslee has written a very systematic and somewhat lengthy paper on this subject which we read in the *New York Medical Journal.*

He divides the subject in endometrical injection, and ingestion.

As is well known, the injection of simple water into the uterus may cause very appalling symptoms; pain, faintness and collapse. This effect is brought about by the coldness of the fluid sometimes used, which of itself may cause painful contractions; by the sudden and forcible distention of the uterus; from a certain hyperæsthesia of the mucous membrane of the urine cavity; and the fluid, it is said, may pass through the fallopian tubes into the peritoneal cavity causing peritonitis.

All bad effects from injections may be guarded against by adopting these precautions:

1. Let the water be at blood-heat or a little less.
2. Introduce it slowly and carefully so as not to disturb the uterine cavity. Only ten or fifteen drops are needed to fill this in a virgin, and twenty-five to forty in one who has borne children.
3. Be sure to provide for a return of the overplus of water by the side of the instrument. This implies a previous dilatation of the cervical canal to a diameter greater than the tube of the syringe.
4. Abstain from the use of all injections, if on introduction of the sound into the uterine cavity, there is found to be tenderness near the fundus, for, in such a condition, even the contact of warm water, will often cause intense agony. If there is no special sensibility of the endometrium, injections, with the precautions enumerated, may be regarded as safe.

Ingestion, or the carrying of a medicinal agent directly to the seat of disease, as paint is applied over the surface to be painted, as usually practiced, has no advantages over injections. By use of proper means it may be made more efficacious than injections, and almost, if not perfectly safe, when applied in the proper conditions.

It is well-known that while simple water or solutions, forced into the uterus, may cause severe symptoms, very strong and stimulating ointments will not. The reason of this is that the latter does not distend the organ ; if enough were used for this purpose the bad symptoms would result. It is usually applied on cotton, and only touches the diseased surface ; if only fifteen or twenty drops was ever used, there would never be any injury done.

Ingesta seems then not to be open to the same objections as the other method. "I advocate this method in all cases in which it is appropriate in preference to injections, and especially when strong applications are to be made."

Of solid ingesta, the nitrate of silver has been used. It has proved an unsafe remedy in some instances ; it has done much damage at times, and doubtless has no advantage over milder applications.

The writer inclines not soon again to use it.

The ointments have, as applications to the endometrium, no advantage over fluids, and may be entirely displaced in practice by the latter form of medication.

The fluid ingesta are of most importance ; they are the same in substance as the injecta.

Endometrical applications are appropriate in metrorrhœa (uterine catarrh) and metrorrhagia. Of course, if either of these is due to displacement of the uterus, or to the presence of tumors, the cause should be first moved if possible.

In metrorrhœa, before applying the medicine, the membrane should be thoroughly cleansed of all secretion. This may be done by injection, but ingestion is quite as effectual, and far more safe.

Ingestion of the medicament is preferable to injection ; is preferable for the same reason.

In metrorrhagia, the blood may properly be removed by injection, and the styptic application to the diseased surface be made by ingestion. Injection in these cases, if properly made, is a perfectly safe procedure.

Endometrical ingestion is required in few of the cases the gynæcologist has to treat ; while injection should far more seldom be resorted to, and never unless preliminary to ingestion of curative agents, in cases where there is no other way to remove blood or secretion, or in cases of metrorrhagia, where styptics are required.

For cleansing the endometrium, a solution of salt is most valuable, and if there is no special sensitiveness a very little soap may be added.

For medicaments, the tincture of iodine is most valuable in metrorrhœa, being used at first ʒ ss to ℥ i of water, then ʒ i to ℥ i, and so on, up to the full strength, if needed.

The sulphate of zinc may be used grs v. to ʒ i of water at first, and then the nitrate of silver grs, v. to ʒ i to the ℥ i of water; the tannic acid from ℈ i to ʒ i to ℥ i of glycerine, and chloride of zinc ʒ ss to ℥ i of glycerine.

If chromic acid is used, it should be with great caution, and not over one part to ten of water used at first.

In metrorrhagia, the per sulphate or per chloride of iron, bears the palm.

To dilate the cervical canal preparatory to injection or ingestion, the sponge tent is the proper method if the cervix is firm and indurated. If the cervix is, however, lax and easy of dilatation, Prof. P. has found very satisfactory a set of steel dilators of his own devising.

There are five dilators in the set, from one-eighth to five-sixteenths of an inch in diameter. Each has a bulb one and three-quarter inches from its point, so that it can pass only that distance into the uterus, and therefore projects less than half an inch into its proper cavity. The instrument may be passed without the use of the speculum, and often the full dilatation may be accomplished at one visit. The dilatation should be carried to at least three-eighths of an inch, whether the fluid is to be injected or ingested.

To accomplish the reflux of fluid in injection, the Professor has devised a tube with conical extremity, three-eighths of an inch in diameter and two inches long. On the sides of the cone are three fenestra, half an inch long each; there is also a fine opening at the end of the tube. When this is inserted the fenestra open into the cavity of the uterus. The fluid is injected through one of the fenestra by means of a syringe with a nozzle perforated at its sides, but not extremity; it flows out readily through the fenestrated openings.

In intra.uterine ingestion, in order to be sure of carrying the remedy and applying it directly to the diseased surface, Prof. Peaslee has devised a tube one and three quarter inches long, and with only two fenestræ. This tube being passed into the uterine cavity, the medicament is carried through the fenestræ and painted over the whole of the endometrium at will, by a mass of cotton. —*Chicago Medical Examiner.*

